# Blood Lead in Children: Laidlaw et al. Respond

**Published:** 2006-01

**Authors:** Mark A. S. Laidlaw, Howard W. Mielke, Christopher R. Gonzalez, Gabriel M. Filippelli, David L. Johnson

**Affiliations:** School of Population Health, University of Western Australia, Crawley, Western Australia, E-mail: mlaidlaw@sph.uwa.edu.au; College of Pharmacy, Xavier University of Louisiana, New Orleans, Louisiana; Department of Geology, Indiana University–Purdue University, Indianapolis, Indiana; State University of New York, College of Environmental Science and Forestry, Syracuse, New York

Our article (Laidlaw et al. 2005) is about seasonality of blood lead (BPb) and developing a predictive model using climatic variables. It is a new and unique finding about lead, marked particularly by the fact that it identifies diffuse soil lead as a significant component of lead sources in urban children. In the article, we argued that meteorologic factors can be robustly applied as a predictor for seasonal variations in children’s BPb, which can be used as a potential tool for health care clinicians in the fight to eliminate lead poisoning in youth. We did not intend to review the literature on lead sources; a vast literature already exists of such studies. Suggesting that we propose the “soil-only hypothesis” completely misconstrues our work.

Brown and Jacobs fail to appreciate our argument that lead accumulation in soil is from a combination of lead-based paint, leaded-gasoline, and many other sources, but is clearly not from lead-based paint alone. They fail to understand the fact that soil normally contains very small amounts of lead, and research from many cities has shown that there has been an excessive accumulation of lead in inner-cities (Filippelli et al. 2005; Mielke 2005). We acknowledge that multiple exposure routes for lead exist for children and likely influence the observed seasonality trends in BPb levels (Laidlaw et al. 2005). We seek to assist with scientific understanding of how and why inner-city children are commonly excessively exposed to lead, and we seek a solution to that problem.

We are concerned that people working at agencies that should champion the reduction of lead exposure do not appreciate the fact that multiple sources of lead have accumulated in urban environments and that all major sources and reservoirs need full attention if we expect to meet the goals of Healthy People 2010 (2005). Our work suggests that, to fully address the childhood lead exposure problem in the United States, a paradigm shift is required that includes all major reservoirs of active lead dust.
